# A review and analysis of new Italian law 219/2017: ‘provisions for informed consent and advance directives treatment’

**DOI:** 10.1186/s12910-019-0353-2

**Published:** 2019-03-04

**Authors:** Marco Di Paolo, Federica Gori, Luigi Papi, Emanuela Turillazzi

**Affiliations:** Section of Legal Medicine, Department of Clinical, Medical, Molecular Pathology and Critical Medicine, University of Pisa, Via Paolo Savi 57, 56126 Pisa, Italy

**Keywords:** Italian law, Informed consent, Advance directives, Conscientious objection, Patients’ self-determination, Shared care planning

## Abstract

**Background:**

In December 2017, Law 219/2017, ‘Provisions for informed consent and advance directives’, was approved in Italy. The law is the culmination of a year-long process and the subject of heated debate throughout Italian society. Contentious issues (advance directives, the possibility to refuse medical treatment, the withdrawal of medical treatment, nutrition and hydration) are addressed in the law.

**Main text:**

What emerges clearly are concepts such as quality of life, autonomy, and the right to accept or refuse any medical treatment – concepts that should be part of an optimal relationship between the patient and healthcare professionals. The law maximizes the value of the patient’s time to decide. Every patient is allowed to make choices for the present (consenting to or refusing current treatment) as well as for the future, conceived as a continuation of the present, and to decide what comes next, based on what he/she already knows. The law identifies three distinct but converging paths towards the affirmation of a care relationship based on reciprocal trust and respect: the possibility to consent to or refuse treatment, the shared care planning, and advance directives.

**Conclusions:**

The fundamental point to emerge from the new Italian law is that consensus is an essential connotation of the treatment relationship. Consensus is not limited to the acceptance/rejection of medical treatment but is ongoing. It is projected into the future through shared care planning and advance directives which act as tools for self-determination and the manifestation of the beliefs and preferences of persons unable to express their will. These principles are in line with the idea of appropriate care as evaluated from two different perspectives, one of scientific adequacy and the other commensurate with the individual’s resources, fragility, values, and beliefs. Surely, however, the new law is not the end of the matter on issues such as conscientious objection, which is deeply rooted within the Italian cultural and political debate. In this regard, healthcare institutions and policymakers will be called upon to develop and implement organizational policies aimed at the management of foreseeable conscientious objection in this field.

## Background

In December 2017, Law 219/2017, ‘Provisions for informed consent and advance directives’ was definitively approved in Italy [1]. Law 219/2017 is the culmination of a year-long process and the subject of heated debate throughout Italian society [2–10].

Increasingly prominent in the public debate in Italy are issues such as advance directives, intended to give autonomous individuals some measure of control over their healthcare strategies even when they have lost the capacity to make their own decisions, the possibility to refuse medical treatment, even when it is lifesaving, and the withdrawal of any medical treatment, and nutrition and hydration [2–10].

On the one hand are those who support the full rights of every individual to make autonomous choices regarding any kind of medical treatment without any time limit. This means that the principle of autonomy and self-determination operates both when patients have the capacity to act and make momentous decisions, as well as in anticipation of future events when this decisional capacity might be lost. On the other hand, there are those who believe that the right of autonomy has some limitations, and hence ethical dilemmas arise. Physicians may be considered as having a duty to preserve the patient’s life even against the latter’s will, preferences, and values.

This cultural debate has long animated the Italian scene and, over the years, numerous bills have been proposed without ever becoming law. Finally, in December 2017 the law was approved by the Italian Parliament. In this article, we describe and analyse the new Italian law, providing a comprehensive view of the general principles behind it and, subsequently, focusing on some specific points of the law itself.

## The new law: Time to decide

The law approved in December 2017 presents a series of issues that seem destined to deeply affect the substance of the care relationship. It states, plainly and indisputably, several principles that deserve our closest attention.

What emerges clearly from the law are concepts such as quality of life, autonomy, and the right to accept or refuse any medical treatment – concepts that should be part of an optimal relationship between the patient and healthcare professionals. The greatest merit of the law is that it maximizes the value of the patient’s time to decide. According to the new law, every patient can and should have the time to make a decision which will prevail even in the future when choice may be partially or wholly prevented by illness. The law recognizes that every patient has the right to have adequate time (subjective, situational, and interpretative) to discuss healthcare-related values, goals, and preferences with physicians, so as to have the chance to make a decision on health treatment which may be current or future, and foreseeable or otherwise. Every patient is allowed to make choices for the present (consenting to or refusing a current treatment) and for the future, conceived as continuation of the present, and to determine what comes next, based on what he/she already knows.

Conclusively, the law has the merit of acknowledging and giving legislative body to the autonomous (i.e., a patient with decisional capacity) patient’s right to make his or her own healthcare-related decisions. This is both in the temporal context of clinical necessity through the tool of consent/refusal regarding diagnostic and/or therapeutic treatments and in anticipation of future events through the tools of shared care planning and advance directives (ADs).

This seems to be the guiding thread of the law which, through its eight articles, affirms the principle of respect for patient autonomy and freedom of choice in all its possible variations (Table 1).

**Table 1:** The essential structure of law 219/2017

Article	Name	Purpose
Article 1	Informed consent	To establish clear rules on consent to and refusal of any medical treatment. To establish the form of informed consent. To establish the role of public and private healthcare facilities in the training of healthcare professionals in the field of relationships and communication with the patient.
Article 2	Pain therapy, prohibition of unreasonable obstinacy in treatment and dignity at the end of life	To avoid non-beneficial treatments and disproportionate means in end-of-life care.
Article 3	Minors and incompetent patients	To establish rules on the decision-making process in the case of minors and incompetent patients.
Article 4	Advance treatment directives	To establish rules on the value of prior requests placed by patients before becoming incapable of expressing their will
Article 5	Shared care plans	To improve the concept of the clinical relationship between patients and healthcare professionals
Articles 6–8	Administrative articles	

## Specific points

The scenario outlined by the law highlights the value of the doctor-patient relationship. The importance of care and trust between patient and doctor is evident from Article 1 (paragraph 2). This is based on the principle of respect for a patients’ autonomy in which his/her decisional capacity and the doctor’s professional autonomy and responsibility are closely intertwined.

It seems to be highly significant that, from the outset, the law makes explicit mention of the concept of trust on which the physician-patient relationship is established. And again, the care relationship opens up to involve, where the patient wishes, also ‘*family members or a partner in civil union or cohabitant or a person the patient trusts’*. In this care relationship, the principle of self-determination of the duly informed patient is reaffirmed. The law identifies three distinct but converging paths towards the affirmation of a relationship based on reciprocal trust and respect: the possibility to consent to or refuse treatment (article 1), the shared planning of treatment (article 5), and advance treatment directives (article 4).

### Informed consent

In article 1 (Informed Consent) the law states that ‘*no medical treatment can be initiated and continued without the free and informed consent of the person concerned, except in cases expressly provided for by l*a*w*’.

Explicit reference is made to constitutional and fundamental rights; thus the full scope of therapeutic self-determination is acknowledged, up to a person’s right to live all the stages of life without undergoing health treatments contrary to his/her will. Consistent with the principle of informed consent as the (normal) legitimation and foundation of health treatment, paragraph 6 of the same article also establishes that ‘*The doctor is obliged to respect the wish expressed by the patient to refuse health treatment or to withdraw from it and, as a result, is exempt from civil or criminal liability*’.

One of the main advantages of the law is that it provides a sort of ‘certification’ of a fundamental principle: in the case of a patient’s refusal of any treatment, or even a request for the physician to withdraw, the doctor’s conduct is ratified as legitimate as a guarantee of the patient’s right. In Italy, in recent years, there has been an impressive increase in medical malpractice claims focusing on the question of informed consent and this has substantially influenced medical practice [11, 12]. Consent has become one of the cornerstones of malpractice lawsuits [13, 14], and in the absence of a clear legislative definition, fear of litigation may contribute to an unjustified approach or defensive practices, and an increased risk of the doctor not respecting the patient’s will to avoid the risk of judicial litigation. The law clearly states that when patients or their legally authorized representatives refuse recommended medical treatments, clinicians are shielded from malpractice lawsuits and criminal charges for respecting the patient’s will.

### Communication time

Some further steps of the law help to create the care relationship within which the patient’s decisional autonomy is implemented.

In article 1 (paragraph 8) the law, moving on a provision already present in the code of medical deontology, states that ‘*Communication time between doctor and patient constitutes treatment time*’ (1, paragraph 8).

It is widely accepted that communication and care are closely intertwined [15] and the importance of communication and conversations between patients and healthcare professionals is widely recognized because of their contribution to quality of care [16–19]. Giving patients enough time is essential to high-quality care [20–22] as communication directly relates to the care provided by doctors themselves. Conversation can be therapeutic as a health care professional who validates the patient’s feelings or expresses empathy may help to improve the patient’s psychological wellbeing and positive emotions (e.g. hope, optimism, self-worth), and diminish the negative ones (e.g. fear, anxiety) [23]. A growing body of evidence indicates that targeted interventions, tailored to the local context, can enhance communication between patients and healthcare professionals in ways that improve patient satisfaction and health outcomes [24–26]*.*

Communication time as treatment time becomes, therefore, a regulatory precept as well as an ethical one that places people, their humanity and their relationships at the centre of medicine. The new law is part of this trend and requires that public and private health structures are organized in such a way as to guarantee patients the necessary information and the adequate training of healthcare professionals [27].

This is, therefore, a fundamental aspect of the law: all healthcare professionals should be trained in communicating so as to be able to ensure that every patient has adequate responses at any time, both during the active phases of treatment and up to the final moments, to guarantee closeness and humanity as well as competence and professionalism. Conclusively, the law implies that health organizations develop and/or implement training programmes designed to improve the knowledge and communication skills of health professionals, using currently available resources for meaningful patient engagement.

### Shared care planning

Closely related to the underlying idea that permeates the law, and that is the enhancement of the care relationship, is the provision of article 5 (shared care planning).

Shared care planning is a dynamic process aimed at ensuring patient engagement in healthcare trajectory across time and practice settings [28, 29]. Their goal is to realize a patient – centered care in which especially individuals with complex medical and social needs may receive treatments in accordance with their values, needs, and preferences [30, 31].

The law thus takes note of the consolidated acquisition of medical practice and translates it into regulations. In the case of chronic diseases or those characterized by an inevitable poor prognosis, *‘shared care planning can be carried out between the patient and the physician*’. Such planning takes into account the possible evolution of the disease, the clinical possibilities to intervene, the patient’s life expectancy in terms of quantity and quality and the planning by which health professionals ‘*are obliged to abide*’ if the patient tends towards a condition of incapacity. Health professionals and patients thus stand on the same side and prepare, together, for the end of life. We can say that article 5 represents an appreciable attempt to strengthen the therapeutic relationship between healthcare professionals and patients, especially in the current context when diseases which have a more protracted dying process are significantly increasing and most deaths are the result of non-sudden events or diseases. New medical treatments and technologies mean that the number of people seeking long-term care is increasing and in high income countries the majority of people die in hospitals, from which it follows that they may have numerous opportunities for dialogue and communication with their physicians about the last period of their life [32].

A further observation, which we consider extremely important in light of the above reflections, focuses mainly on the subject of shared decision making. This is the concept of appropriateness of care that seems to emerge from the new law and that is attuned to both a technical-scientific point of view and to a much more subjective one, relating to the patient’s needs, desires and fragility. In fact, to talk about appropriate care in medicine is to address different aspects of the broader concept of appropriateness: evidence-based care, clinical expertise, patient-centeredness, resource use and equity, employed in varying combinations with overlapping themes and subthemes [33]. Also in Article. 2 (Treatment of Pain), the law calls for health professionals to intervene ‘*using means appropriate to the medical situation of the patient*’ and to refrain from any unreasonable obstinacy in the administration of treatment and the use of unnecessary or disproportionate treatments. Furthermore, it refers to the requirement of appropriateness of care in the event of disagreement between the doctor and the representative of the incapacitated person (Article 3, paragraph 5). The law stipulates that, should the legal representative of the incapacitated person, or the support administrator in the absence of an advance directive, or the legal representative of the child, refuse treatment that the doctor considers to be appropriate and necessary, the decision is remitted to the tutelary judge.

The law seems to strengthen the idea of proportionality of care, drawing together, on the one hand, the expertise of clinicians and the scientific evidence and, on the other, the patient’s values, culture, needs, preferences, perceptions and acceptance of care. In other words, the law finally sanctions the change from a concept of medical care where physicians have veered towards a ‘medicalised’ perspective that has been heavily dependent upon clinical judgement to a more comprehensive one that encompasses a wider evaluation of the patient’s interests, of which the medical perspective is but one component, the other being the very personal values of patients themselves [34]. Conclusively, it has to be highlighted that the new Italian law not only promotes patient centeredness, but it also emphasizes respect for patients’ autonomy, enhancing patients’ healthcare values, goals and preferences and incorporates them into care plans.

### Advance directives

Finally, at the end of a very long and difficult debate in Italy surrounding all the previous bills [2–10], article 4 of the new law is dedicated to the issue of ADs.

Article 4 (paragraph 1) establishes that ‘*every competent person, in preparation for a possible loss of the capacity for self-determination in the future, and after having acquired adequate medical information on the consequences of his/her choices, can, through the advance directives, express his/her own will regarding health treatments, as well as consent or refusal with respect to diagnostic tests or therapeutic choices and to individual health treatments. He/she can also choose a ‘trustee’ to take his/her place and represent him/her in the relationship with healthcare professionals and organizations*’.

ADs appear as an expression – different only with regard to temporal collocation – of that consensus between healthcare professionals and patients which is the main theme of the law. In other words, we can say that ADs express their effects in a time window which is different from consent/refusal regarding current treatment. Should patients not be in a position to competently decide how they ought to be treated and what kind of treatment could be deemed desirable or dignified, ADs allow them to plan accordingly.

Their function is, therefore, to preserve the will of the informed patient also in the future, thus abiding by the principle of equality for one of the most important ‘new rights’: the right to informed consent/dissent. ADs ought to be viewed as an extension of the fully autonomous person.

The law does not specifically stipulate what can and cannot be included in an AD. However, article 4 refers to that expressed in the introductory article on informed consent, evoking the general principle that a patient cannot demand treatments contrary to the law, professional ethics, or to good clinical practices. If faced with such requests physicians have no professional obligations.

The provision referred to in article 4, that whoever wishes to draft/write ADs should first acquire ‘*adequate medical information on the consequences of his/her choices*’, ensures, opportunely, that the advance decision is not the result of inaccurate, incomplete, outdated or even unmedical/unscientific information. However, the law does not specify if medical information should come from a doctor and, if so, what kind of doctor (generalist or specialist), or if the autonomous retrieval of information by the patient is sufficient. Given the fact that many patients use the Internet and many other sources to obtain information and familiarize themselves with medical conditions [35], the risk is that uncontrolled sources of medical information could undermine the adequacy and real awareness of the choices made in the ADs. In fact, potential obstacles facing individuals searching for medical information from several different sources might be: accessing the desired information, assessing the quality of the information found, and applying the information in the person’s life [36–38].

This presents a great challenge for the Italian medical body which, by law, is called on to retrieve this role. In fact, only the physician appears to be fully able to provide ‘adequate’ medical information to the patient who wants to formulate ADs. Account must be taken of the fact that this entails deciding on a very wide range of pathological and healing hypotheses, since planning necessarily takes place when there is not yet a specific disease or a defined therapy. Once again, the professionalism and the role of doctors in their relationship with patients and citizens is highlighted in the article on ADs.

### Conscientious objection

The new Italian law does not explicitly mention the possibility of conscientious objection (CO) for healthcare professionals caring for patients who have expressed their will regarding medical treatments. Indeed, the law states (art. 5, paragraph 5) that ‘the physician is required to respect the ADs’, meaning that it is legally binding and not simply advisory. Only when ADs appear to be clinically inappropriate is the physician legitimated to disregard them. In fact, in the same article 4 it is established that *‘[...] the doctor takes into account the ADs, which may be disregarded, in whole or in part, by the same doctor, in agreement with the trustee, if they clearly appear incongruous or do not correspond to the current clinical condition of the patient or if there are therapies which were unforeseen at the time of writing which would offer sound possibilities for improved living conditions*. [...]’. Furthermore, as mentioned above, the law evokes the general principle that ‘*the patient cannot demand health treatment contrary to the law, professional ethics or good clinical-care practice; with regard to such requests, the doctor has no professional obligations’.*

Following the enactment of the law, a heated national debate has arisen on the issue of the right to CO on the part of health professionals who care for patients who have drawn up ADs. This is part of an international debate on the same theme [39–51].

One of the thorniest issues concerning CO is how to balance two potentially conflicting situations: the physician’s right to freedom and the patient’s wishes and interests. It could be said that physicians have the right to object on conscientious grounds; however, this right cannot be considered absolute as it should not compromise patients’ rights, values, needs, and priorities [39]. Further potential limits to the physician’s right to CO arise when CO would result in the harassment of patients themselves [39]. Failure to abide by ADs that are the result of considerable thought and suffering, shared by the patient and a health professional or team, could mean the imposition of treatment that is held by the patient to be undesirable, inappropriate and/or undignified. It would thus undermine the patient’s best interests and quality of life.

In this international debate, some additional information is necessary to delineate the Italian climate.

Strong legal grounds exist in Italy for CO since the Italian Constitution, through several articles, recognizes and guarantees a sort of ‘freedom of conscience’ as an inviolable human right, protecting freedom of religion, and of expression and thought. Furthermore, a specific conscience clause is included in several deontological codes for healthcare professional categories that recognize a broad right to CO for healthcare workers. Finally, professional associations of health workers often defend the right of their members not to be forced to carry out interventions that are contrary to their moral beliefs [52]. Italy is a Catholic country where questions concerning CO are deeply rooted in the cultural and political debate [53, 54]. Following approval of the law, there were numerous attacks from the Italian Catholic world, focusing especially on the part which does not foresee CO as a possibility for physicians, health workers and institutions [55, 56]. Detractors of the law claim that it is strongly biased towards the autonomy of the patient and leaves no room for decision making to the doctor for whom no form of CO is recognized. It is argued that CO is a fundamental human right guaranteed by the Italian Constitution and by the Italian code of medical ethics and that the lack of explicit provision for it is an insurmountable weakness of the law. The risk could be to reduce the role of healthcare workers to that of mere executors of the patient’s will [52].

Taking a different position are those who support the idea that the right to CO is not absolute and can be overridden by limitations aiming to protect both public interests and the rights and freedom of patients. Conflicting interests, such as the protection of clinicians’ moral integrity and respect for their autonomy, as opposed to protection of and respect for patients and the need to avoid discrimination, are at stake.

Beyond the discussion as to whether CO to advance directives could be morally acceptable and/or legally permissible, the core question confronting Italian institutions is how to allow patients to ensure their legitimate ADs are respected while still allowing healthcare personnel the option of CO.

Health professionals, hospital administrators and Italian policymakers will inevitably be called on to manage CO in intensive care and shared care planning settings. Institutions could be called on to ensure that clinicians and members of the treatment team, other than the objector, are prepared to support the patient’s decision to withdraw from or not to initiate treatment, and are available and willing to take over care.

This could allow CO to be exercised by an individual clinician without compromising the patient’s right to have their wishes concerning health treatments fulfilled. In healthcare institutions, especially public ones, organizational policies will be necessary to guarantee the conditions under which the rights of both groups can be exercised.

Undoubtedly, public health structures have a duty to guarantee that all citizens can not only take advantage of all medical services and treatments guaranteed by law, but also, in the light of the new legislative provisions on shared care planning and ADs, opt out of unwanted treatment. Whether or not private hospitals should be able to choose what kind of treatment to offer their patients might be open to discussion; however, publicly funded hospitals have different duties and responsibilities from private ones [53, 57].

It clearly emerges how this form of accommodation could create significant financial or additional organizational burdens and costs for the institution, such as increasing the available personnel, to guarantee an ideal ratio of conscientious objectors to non- objectors [53, 57].

## Conclusions

In Italy, recent developments in the cultural, juridical, and social debate have paved the way for a law which insists on a more patient-centred standard of medical care in the best interests of the patients and which is an attempt to regulate all the complex issues surrounding end-of-life care.

This article highlights the fundamental point of the new Italian law: consensus as an essential connotation of the treatment relationship. This is not limited to the acceptance/rejection of a medical treatment but expands in time and is projected into the future through shared care planning and ADs which act as tools of self-determination and the manifestation of the beliefs and preferences of someone who is unable to make decisions.

These principles are perfectly in line with the idea of appropriate care, as evaluated from two different perspectives, one of scientific adequacy and the other commensurate with the individual’s resources, fragility, values, and beliefs.

The operational challenges in achieving this normative goal still remain, among which is the goal of balancing the clinician’s right to conscientious objection and the patient’s right to self-determination. These challenges deserve the close attention of Italian healthcare professionals and policymakers.

## Abbreviations

ADs: Advance directives
CO: Conscientious objection
